# Prescription of Chinese Herbal Medicine in Pattern-Based Traditional Chinese Medicine Treatment for Depression: A Systematic Review

**DOI:** 10.1155/2015/160189

**Published:** 2015-06-09

**Authors:** Wing-Fai Yeung, Ka-Fai Chung, Ka-Yan Ng, Yee-Man Yu, Shi-Ping Zhang, Bacon Fung-Leung Ng, Eric Tat-Chi Ziea

**Affiliations:** ^1^School of Chinese Medicine, University of Hong Kong, Hong Kong; ^2^Department of Psychiatry, University of Hong Kong, Pokfulam Road, Hong Kong; ^3^School of Chinese Medicine, Hong Kong Baptist University, Hong Kong; ^4^The Chinese Medicine Department, Hospital Authority, Hong Kong

## Abstract

Traditional Chinese medicine (TCM) treatments are often prescribed based on individuals' pattern diagnoses. A systematic review of randomized controlled trials in Chinese and English literatures on TCM pattern-based treatment for depression has therefore been conducted. A total of 61 studies, 2504 subjects, and 27 TCM patterns were included. Due to the large variation of TCM pattern among participants, we only analyzed the top four commonly studied TCM patterns: *liver qi depression, liver depression and spleen deficiency, dual deficiency of the heart, and spleen* and *liver depression and qi stagnation*. We found that Xiaoyao decoction was the most frequently used herbal formula for the treatment of *liver qi depression* and *liver depression with spleen deficiency*, while Chaihu Shugan decoction was often used for *liver depression and qi stagnation*. Bai Shao (*Paeonia lactiflora* Pall.) and Chai Hu (*Bupleurum chinense* DC.) were commonly used across different TCM patterns regardless of the prescribed Chinese herbal formulas. The rationale underlying herb selection was seldom provided. Due to the limited number of studies on TCM pattern-based treatment of depression and their low methodological quality, we are unable to draw any conclusion regarding which herbal formulas have higher efficacy and which TCM patterns respond better to CHM.

## 1. Introduction

According to the Global Burden of Disease Study 2010, major depressive disorder (MDD) was ranked the second leading cause of years lived with disability, accounting for 8.2% of all years lived with disability [1]. The World Mental Health Survey Initiative showed that the average lifetime prevalence for major depressive episode based on the Diagnostic and Statistical Manual, Fourth Edition [2], was 14.6% in 10 high-income countries and 11.1% in 8 low- to middle-income countries [3]. Pharmacotherapy is currently the most commonly used treatment for MDD because of its reported effectiveness; however, complaints such us nausea, headache, insomnia, agitation, weight gain, daytime somnolence, and sexual dysfunction are common during the course of treatment, leading to early termination in some patients. The use of psychotherapy as an alternative is no better because of its time-intensive nature, limited access to skilled providers, high cost, and requirement of patients' participation and motivation. Faced with the limitations of the currently available treatments, complementary and alternative medicine for depression is very common. A national representative survey in the United States found that 53.6% of people with depression reported using some forms of complementary and alternative therapies to treat depression during the past 12 months [4].

Chinese herbal medicine (CHM) is one of the oldest medical treatments in the world and it is a common form of complementary and alternative medicine therapy for MDD [5, 6]. Previous studies have been conducted to examine the efficacy of CHM for depression [7–14]; however, limited information is available on pattern-based CHM treatment. According to the traditional Chinese medicine (TCM) theory, eight major parameters,* yin* and* yang*,* external* and* internal*,* hot* and* cold*, and* excess* and* deficiency*, are used to describe the patterns of bodily disharmony. Additional systems, such as* qi*,* blood*, and* body fluid* differentiation and* zang fu* (organ) differentiation are also used [15]. In terms of the TCM theory, the onset of depression is often due to “damages” by extreme emotions.* Liver qi* is first affected, followed by disharmony of the* qi* mechanism of the five viscera, particularly* liver*,* spleen*, and* heart*, resulting in a loss of regulation of the* qi* and* blood*. The* liver* depression may repress the* spleen* and lead to consumption and damage of the* heart qi*. If* heart* loses its nourishment and the restfulness to* heart shen* (spirit) occurs, it will lead to unstable and depressed mood. When* qi* depression is prolonged, it will accumulate and transform into fire [16, 17].

TCM pattern differentiation is a diagnostic conclusion of the pathological changes of a disease state based on an individual's symptoms, physical signs, pulse form, and tongue appearance. Although it is believed that pattern-based TCM treatment will provide better efficacy, previous studies regarding the additional benefits of TCM pattern differentiation are scarce. One randomized controlled trial (RCT) found that the therapeutic effect of Chinese herbal treatment according to TCM pattern was more sustainable than a standard formula in treating irritable bowel syndrome [18]. It has also been reported in rheumatoid arthritic patients that TCM pattern diagnoses can guide the use of Western medicine [19]. To the best of our knowledge, no systematic review has been conducted on pattern-based CHM treatment for depression. Given the high prevalence of depression and its frequent presentation to TCM practitioners, it is important to review the current application of pattern differentiation in CHM treatment for depression. The objectives of this paper were (1) to summarize the commonly diagnosed TCM patterns in patients with depression and (2) to find out the current practice of pattern-based CHM treatment for depression.

## 2. Materials and Methods

The present study was part of our systematic review on Chinese herbal medicine for depression [11, 20]. Two researchers (Ka-Yan Ng and Yee-Man Yu) independently searched nine Chinese language databases (China Journals Full-text Database, China Proceedings of Conference Full-text Database, Chinese Biomedical Literature Database, China Doctor Dissertations Full-text Database, China Master Theses Full-text Database, Chinese Science and Technology Documents Database, Chinese Dissertation Document Bibliography Database, Taiwan Electronic Periodical Services, and WanFang Database) and seven English language databases (MEDLINE, EMBASE, Cochrane Central Register of Controlled Trials, Cumulative Index to Nursing and Allied Health Literature, Allied and Complementary Medicine, PsycINFO, and ProQuest Dissertations and Theses A&I) using the grouped terms “depression^*∗*^ or depressive^*∗*^ or dysthymia^*∗*^ or mood disorder^*∗*^ or “affective disorder^*∗*^” or “affective symptoms” or MDD” and “Chinese herb^*∗*^ or herbal medicine^*∗*^ or traditional Chinese medicine^*∗*^ or TCM or Chai-Hu-Shu-Gan-San or ChaiHuShuGan^*∗*^ or Xiao-Yao-San or XiaoYao^*∗*^ or Ban-Xia-Hou-Pu-Tang or BanXiaHouPu^*∗*^ or Gan-Mai-Da-Zao-Tang or GanMaiDaZao^*∗*^ or Gui-Pi-Tang or GuiPi^*∗*^ or Wen-Dan-Tang or WenDan or Yue-Ju-Wan or Yue-Ju” and the equivalent Chinese terms. We imposed no language restriction. We also checked the reference lists of the included papers and previous systematic reviews [7–14] for relevant articles.

### 2.1. Selection Criteria

Studies included in this review were RCTs that described TCM patterns of depressed subjects who received CHM treatment for depression. In order to obtain a full coverage of the topic, we had not set any specification for outcome measure and study quality. In addition, to derive a general picture of TCM pattern utilization, studies were excluded if they (1) had less than 30 subjects; (2) examined male or female only; (3) focused on subjects aged below 18 or above 70 years; (4) focused on a particular life transition period or a specific TCM pattern; (5) had no statistical information regarding the frequency of individual TCM pattern; or (6) were duplicated publications.

### 2.2. Data Extraction Process

Any disagreement about the eligibility of a study was resolved by discussion between the two researchers who independently selected the relevant publications and by consultation with the senior authors (Wing-Fai Yeung and Ka-Fai Chung). One author extracted the data (Ka-Yan Ng) and the other (Yee-Man Yu) checked the extracted data. For each study, the following variables were extracted: study design, sample size, mode of recruitment, sampling and diagnostic procedure, inclusion and exclusion criteria, and participants' characteristics including age, gender, and duration of depression. TCM patterns, treatment principles, treatment regimen and outcome of TCM treatments were obtained. All Chinese to English translations were deduced primarily from the* World Health Organization (WHO) International Standard Terminologies on Traditional Medicine in the Western Pacific Region* [21] and additionally from* Traditional Chinese Internal Medicine* [22], a commonly used English-language TCM textbook in China.

### 2.3. Study Quality Assessment

We assessed the methodological quality using the Jadad scale [23]. Points are awarded if the study is described as randomized, 1 point; has appropriate randomization method, 1 point; is described as double-blind, 1 point; uses appropriate blinding method, 1 point; or has description of withdrawals and dropouts, 1 point. A Jadad scale score ≥3 represents better quality trials.

### 2.4. Statistical Analysis

SPSS version 20.0 was used for statistical analysis. Data were summarized using mean (SD) and 95% confidence intervals (CIs).

## 3. Results

**Figure 1:**
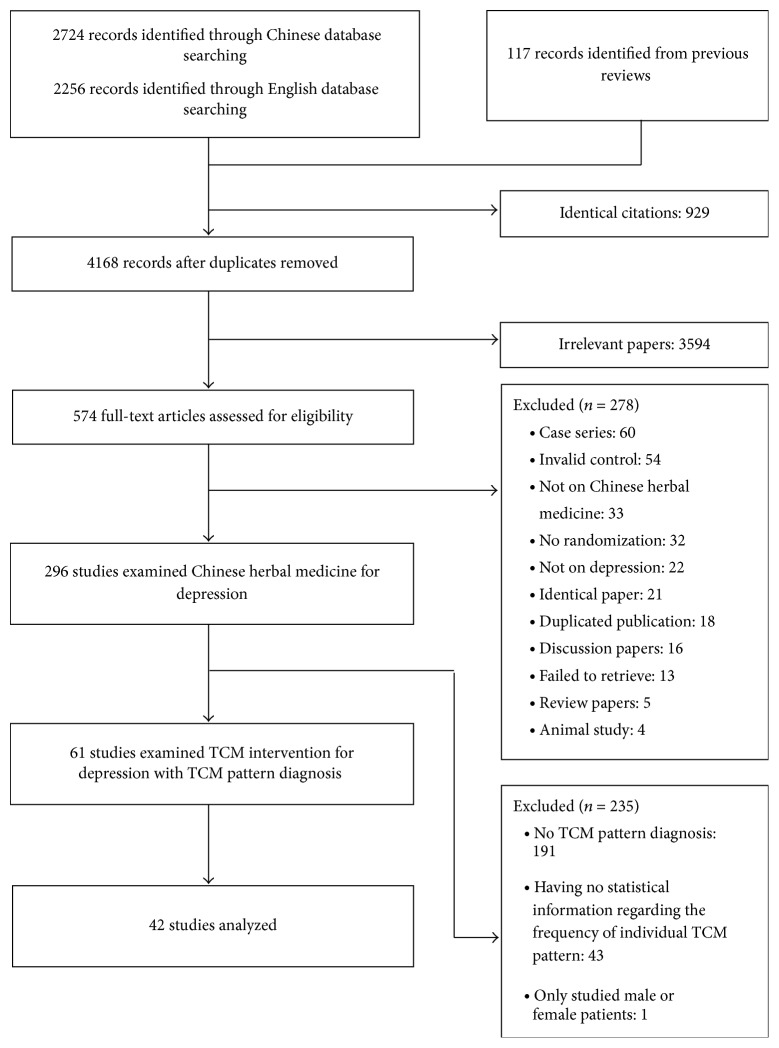
Study selection flowchart.

The search yielded 5097 potential titles for review, of which 929 were duplicated records and 3594 were excluded for reasons of irrelevance. The full text of 574 was retrieved for detailed assessment, of which 278 were excluded for various reasons (Figure 1). Of the 296 studies on CHM for depression, 61 of them examined pattern-based treatment. A total of 27 different TCM patterns were identified in the 61 studies. We analyzed the most commonly studied TCM patterns:* liver qi depression, liver depression and spleen deficiency, dual deficiency of the heart and spleen, and liver depression and qi stagnation* and* liver-kidney yin deficiency*. These four commonly studied TCM patterns were described in 42 of the 61 studies (68.9%) and involved 1762 subjects accounting for 70.4% of the total 2504 subjects (Table 1). Eighteen of the 42 studies examined CHM alone and the other 24 studies examined CHM plus antidepressants [24–65]. The 1762 participants had a mean age of 40.7 years, of which 59.0% were female. The participants were suffering from depression unspecified in 33 of the 42 studies, six studies on poststroke depression and three on depression comorbid with diabetes. The diagnosis of depression was based on 17-item or 24-item Hamilton Depression Rating Scale (HAMD_17/24_) in 36 studies, the Chinese Classification of Mental Disorder Second/Second-revised/Third Edition (CCMD-2/2-R/3) in 35 studies, Zung Self-rating Depression Scale (SDS) in seven studies, and one study each using DSM-IV, Clinical Global Impression Scale (CGI), and International Classification of Diseases Version 10 (ICD-10). The response to intervention was assessed by the HAMD17/24 in 34 studies and by effective rate in 33 studies.

The criteria used for TCM pattern diagnosis were reported in 27 of the 42 studies. The criteria were based on the TCM Syndrome Diagnostic Standard (*N* = 7), New Guidelines for TCM Clinical Research (*N* = 3), TCM Diagnostic Standard for Depression (*N* = 2), Chinese Professional Association of Integrative Medicine Diagnostic Criteria for Mental Disorders, Version 1991/2001 (*N* = 3), Chinese Classification and Diagnostic Criteria of Mental Disorders (*N* = 1), and TCM textbooks (*N* = 13). However, none of the studies described other details of the diagnostic procedure and the background of practitioners who made the pattern diagnosis.

### 3.1. Methodological Quality

Twenty-three (54.8%) of the 42 studies were described as randomized but the randomization method, blinding, and dropouts were not presented; hence, these 23 studies only had a Jadad scale score of one. Seventeen studies (40.5%) had a Jadad scale score of two, and only two studies (4.8%) obtained a Jadad scale score of four.

### 3.2. Pattern-Based CHM Treatment

#### 3.2.1. Liver qi Depression

According to the TCM theory,* liver qi depression* is an impairment of the* liver* function, obstructing free movement of* qi* and resulting in stagnation of* qi* in* liver* [21]. Nineteen studies examined CHM treatment in patients with* liver qi depression*. Seven studies used CHM alone and 12 used CHM-antidepressant combination. Xiaoyao decoction was investigated in 13 (68.4%) of the 19 studies, while other CHM formulas were studied in only one to two studies. Seventeen studies reported the ingredients of the CHM formulas for the treatment of* liver qi depression*. Chai Hu (*Bupleurum chinense* DC.) was the most commonly used herb, followed by Bai Shao (*Paeonia lactiflora* Pall.), Dang Gui (*Angelica sinensis* (Oliv.) Diels), Fu Ling (*Poria Cocos* (Schw) Wolf.), and Bai Zhu (*Atractylodes macrocephala* Koidz.). These five herbs were chosen for treating* liver qi depression* in more than half of the 17 studies that had reported the CHM ingredients (Table 2). The mean effective rate of Xiaoyao decoction for the treatment of* liver qi depression* was 84.7% and the mean HAMD change score was 19.1 (Table 3); for Xiaoyao decoction-antidepressant combination for* liver qi depression*, it was 86.0% and 19.1, respectively (Table 4). Tables 3 and 4 present the overall efficacy of pattern-based CHM monotherapy and CHM-antidepressant combination for* liver qi depression*.

#### 3.2.2. Liver Depression and Spleen Deficiency

According to the TCM theory,* liver depression and spleen deficiency* is a pathological change in which the transporting and transforming function of the* spleen* is affected by depressed* liver qi*, leading to* spleen* deficiency [21]. Of the 13 studies on* liver depression and spleen deficiency*, three studies examined CHM alone and 10 investigated CHM-antidepressant combination. The most frequently used CHM formula was also Xiaoyao decoction, which was used in 9 of the 13 studies. Nine of the 13 studies provided ingredients of the CHM formulas. The commonly used single herbs for the treatment of* liver depression and spleen deficiency* were Bai Shao (*Paeonia lactiflora* Pall.), Fu Ling (*Poria Cocos* (Schw) Wolf.), Chai Hu (*Bupleurum chinense* DC.), Zhi Ke (*Citrus *×* aurantium* L.), Dang Gui (*Angelica sinensis* (Oliv.) Diels), and Bai Zhu (*Atractylodes macrocephala* Koidz.) (Table 2). Only one study was conducted on Xiaoyao decoction monotherapy for* liver depression and spleen deficiency*, and the HAMD change score was 7.3 (Table 3). The mean effective rate of Xiaoyao decoction-antidepressant combination for* liver depression and spleen deficiency* was 86.0% and the mean HAMD change score was 20.1 (Table 4). Tables 3 and 4 show the overall efficacy of pattern-based CHM monotherapy and CHM-antidepressant combination for* liver depression and spleen deficiency*.

#### 3.2.3. Dual Deficiency of the Heart and Spleen

*Dual deficiency of the heart and spleen* is a condition in which both* heart blood* and* spleen qi* are deficient, leading to disordered* heart* function and an impairment of the transporting and transforming function of* spleen* [21]. There was significant variation in the TCM pattern-based CHM treatment for* dual deficiency of the heart and spleen*. Of the 10 relevant studies, seven different CHM formulas were used. Guipi Tang and Ningcao Wangyou Tang were each used in two studies, while other CHM formulas were only examined once. The frequently used single herbs for* dual deficiency of the heart and spleen* were Gan Cao (*Glycyrrhiza uralensis* Fisch.), Bai Shao (*Paeonia lactiflora* Pall.), Chai Hu (*Bupleurum chinense* DC.), Fu Ling (*Poria Cocos* (Schw) Wolf.), Bo Zi Ren (*Platyclatus orientalis* (L.) Franco), Da Zao (*Ziziphus jujua* Mill.), and Yu Jin (*Curcuma wenyujin* Y. H. Chen & C. Lin) (Table 2). The mean effective rate of CHM monotherapy for* dual deficiency of the heart and spleen* was 91.5% and the mean HAMD change score was 10.8 (Table 3); for CHM-antidepressant combination, it was 77.0% and 11.4, respectively (Table 4).

#### 3.2.4. Liver Depression and qi Stagnation

*Liver depression and qi stagnation* is a pathological change in which* liver* is depressed, leading to impeded circulation of* qi* and stagnation of* qi* movement [21]. The most frequently used CHM formula for* liver depression and qi stagnation* was Chaihu Shugan decoction (Table 3). The commonly used single herbs were Bai Shao (*Paeonia lactiflora* Pall.), Yu Jin (*Curcuma wenyujin* Y. H. Chen & C. Lin), Chai Hu (*Bupleurum chinense* DC.), Chuan Xiong (*Ligusticum striatum* DC.), Xiang Fu (*Cyperus rotundus* L.), Zhi Ke (*Citrus* ×* aurantium* L.), Xiang Fu (*Cyperus rotundus* L.), and He Huan Pi (*Albizia julibrissin* Durazz.) (Table 2). The mean effective rate of CHM monotherapy for* liver depression and qi stagnation* was 90.4% and the mean HDRS change score was 17.5 (Table 3); for CHM-antidepressant combination, it was 80.0% and 15.0, respectively (Table 4).

## 4. Discussion

This study is the first systematic review of English and Chinese literature, involving 61 studies and 2504 subjects on the classification and treatment of depression using the TCM diagnostic system. We found that the TCM pattern diagnoses for depression were diverse. Among 27 different TCM patterns identified,* liver qi depression* was the most commonly diagnosed TCM pattern in people with depression, followed by* liver depression and spleen deficiency*,* dual deficiency of the heart and spleen,* and* liver depression and qi stagnation*. With regard to CHM treatment, Xiaoyao decoction was the most frequently used herbal formula for the treatment of* liver qi depression* and* liver depression and spleen deficiency*, while Chaihu Shugan decoction was often used for* liver depression and qi stagnation*. For* dual deficiency of the heart and spleen*, no single formula could be regarded as commonly used across TCM practitioners. The results suggest that TCM practitioners may be more consistent in the treatment of depression involving* liver depression* than other patterns. The abundance of low-quality studies highlights that knowledge and experience in conducting high-quality RCTs may be limited. It further suggests that institutional review boards and publishing journals should play an active role in monitoring the standards of clinical trials.

The present paper found that Bai Shao (*Paeonia lactiflora* Pall.), which has a function of nourishing the* blood* and emolliating the* liver*, and Chai Hu (*Bupleurum chinense* DC.), which can sooth the* liver*, were commonly used to treat depression regardless of the TCM pattern. Animal studies have found that extract from Bai Shao produces antidepressant effects in chronic unpredictable stress-induced depression model in mice and rats [66]. The antidepressant effects are likely mediated by inhibition of the monoamine oxidase activity and oxidative stress, upregulation of neurotrophins, and modulation of the function of the hypothalamic-pituitary-adrenal axis [66]. Pharmacological studies of Chai Hu have shown that it has hepatoprotective, anti-inflammatory, antipyretic, analgesic, and immunomodulatory effects [67–70]; however, its antidepressant effects remain unclear. According to a TCM textbook, Chai Hu is a “Sovereign” herb in Xiaoyao decoction and Chaihu Shugan decoction [69]. The “Sovereign” herb is used for treating the principal diseases based on the TCM theory; the “Minister” herb has synergistic effects with “Sovereign” herbs and helps to alleviate other accompanying symptoms; the “Assistant” herb is for enhancing the therapeutic effects and modulating the adverse effects of “Sovereign” and “Minister” herbs, while the “Courier” herb is used for harmonizing the actions of the others [66]. Therefore, some of the commonly used herbs identified in this review may not primarily be targeted at depression; instead, they indirectly alleviate depression by enhancing or harmonizing the actions of other herbs. In view of the common use of Chai Hu in the treatment of depression, further studies on its antidepressant effects are warranted.

Xiang Fu (*Cyperus rotundus* L.) and He Huan Pi (*Albizia julibrissin* Durazz.) were specifically used for* liver depression and qi stagnation*, and Bo Zi Ren (*Platyclatus orientalis* (L.) Franco), which has a function of nourishing the* heart* and tranquilizing* shen*, was specific for* dual deficiency of the heart and spleen*. Yu Jin (*Curcuma wenyujin* Y. H. Chen & C. Lin) was commonly used for* liver depression and qi stagnation* and* dual deficiency of the heart and spleen*, Fu Ling (*Poria Cocos* (Schw) Wolf.) for* liver qi depression*,* liver depression and spleen deficiency,* and* dual deficiency of the heart and spleen*, and Dang Gui (*Angelica sinensis* (Oliv.) Diels) and Bai Zhu (*Atractylodes macrocephala* Koidz.) for* liver qi depression* and* liver depression and spleen deficiency*. Since three of the four commonly diagnosed TCM patterns in people with depression involve* liver* depression and two involve* spleen* deficiency, the prescription of Chinese herbs for different TCM patterns are inevitably overlapping. A lack of consistency across TCM practitioners in their selection of herbal formulas and pattern-based prescription of individual herbs may also lead to variation in CHM treatment [70]. Considering the limited number of studies available, further research on pattern-based CHM treatment for depression is warranted.

Due to the studies' variation in study design and inadequate methodological quality, it is difficult to conclude which herbal formulas have higher efficacy and which TCM patterns respond better to CHM. As a whole, there is no evidence to suggest CHM-antidepressant combination has higher efficacy than CHM monotherapy for depression. We found that the effective rate was generally high for pattern-based treatment in* liver qi depression* and was similar between CHM monotherapy and CHM-antidepressant combination. For* liver depression and spleen deficiency*, the efficacy of CHM monotherapy and CHM-antidepressant combination was similar in terms of effective rate, but it was lower with CHM monotherapy in terms of mean HAMD change score (9.7 versus 20.1 for CHM-antidepressants combination). For* dual deficiency of the heart and spleen*, the efficacy of pattern-based CHM treatment was relatively weaker, especially CHM-antidepressant combination, which had a mean effective rate of 77.0% and mean HAMD change score of 11.4. For* liver depression and qi stagnation*, the efficacy of CHM monotherapy and CHM-antidepressant combination was similar, except for a relatively low mean effective rate of CHM-antidepressant combination (80.9% versus 90.4% for CHM monotherapy). It is clear that further studies with better methodological quality are needed to delineate the efficacy of pattern-based CHM treatment in depression.

There are some methodologic limitations of the study. First, there is no “gold standard” in the classification of TCM patterns, and the criteria for each pattern might be different between researchers. Future studies using both Western and Chinese medicine systems in diagnosis and severity assessment may facilitate Western-Chinese medicine integration in the understanding and treatment of depression. Although we reported the pattern diagnosis by the authors, the procedure and quality of the diagnostic process was uncertain. Such uncertainties would inevitably lead to discrepancies in the selection of herbs in treatment. Although a large number of studies were reviewed, this paper only summarized the effective rate and HAMD change score based on RCTs; meta-analysis was not possible due to difference in study design and low methodological quality of the studies.

Despite the limitations, the present study, for the first time, systematically and comprehensively summarized important data on pattern-based CHM treatment for depression. Our data should be useful for both clinical practice and future research. More high quality studies incorporating TCM pattern differentiation and treatment principle are needed to examine the efficacy of TCM treatments and the additional benefit of pattern differentiation.

## 5. Conclusion

We found that* liver qi depression*,* liver depression and spleen deficiency*,* dual deficiency of the heart and spleen*, and* liver depression and qi stagnation* were the most commonly studied TCM patterns in people with depression. In addition, Bai Shao (*Paeonia lactiflora* Pall.) and Chai Hu (*Bupleurum chinense* DC.) were commonly used across different TCM patterns regardless of the prescribed Chinese herbal formulas. Due to the limited number of studies on TCM pattern-based treatment of depression and their low methodological quality, we are unable to draw any conclusion regarding which herbal formulas have higher efficacy and which TCM patterns respond better to CHM.

## Figures and Tables

**Table 1:** The most common TCM patterns diagnosed in people with depression.

TCM pattern	Chinese name	Number of subjects diagnosed with the TCM pattern (%) (total *N* = 1762)	Number of studies that examined the TCM pattern (%) (total *N* = 42)^a^
*Liver qi depression *	*肝氣鬱結*	797 (45.2%)	19 (45.2%)
*Liver depression and spleen deficiency *	*肝鬱脾虛*	425 (24.1%)	13 (31.0%)
*Dual deficiency of the heart and spleen *	心*脾兩虛*	315 (17.9%)	9 (21.4%)
*Liver depression and qi stagnation *	*肝鬱氣滯*	225 (12.8%)	8 (19.0%)

^a^Six studies examined more than one TCM pattern.

**Table 2:** The commonly used Chinese herbal medicine for depression in subjects diagnosed with different TCM patterns.

	*Liver qi depression *	*Liver depression and spleen deficiency *	*Dual deficiency of the heart and spleen *	*Liver depression and qi stagnation *
Number of studies that examined the TCM pattern	*N* = 19	*N* = 13	*N* = 9	*N* = 8
Commonly used Chinese herbal formula (*N*, % of studies that examined the TCM pattern)	Xiaoyao decoction (13, 68.4%)	Xiaoyao decoction (9, 69.2%)	Guipi decoction (2, 22.2%); Ningcao Wangyou decoction (2, 22.2%)	Chaihu Shugan decoction (5, 62.5%)
Number of studies that provided the composition of herbal formula	*N* = 17	*N* = 9	*N* = 8	*N* = 6
Number of studies that provided TCM treatment principle	*N* = 6	*N* = 4	*N* = 3	*N* = 4
Composition of herbal formula (% of studies that provided the formula's composition)^a^				
Bai He [*Lilium brownii* F.E.Br. ex Miellez]	11.8%	/	/	/
Bai Shao [*Paeonia lactiflora* Pall.]	70.6%	100%	50.0%	100%
Ban Xia [*Pinellia ternata* (Thunb.) Makino]	17.6%	11.1%	37.5%	16.7%
Bai Zhu [*Atractylodes macrocephala* Koidz.]	52.9%	55.6%	25.0%	16.7%
Bei Mu [*Fritillaria cirrhosa* D.Don]	/	/	25.0%	/
Bing Pian [*Dryobalanops aromatica* Gaertn. f.]	/	/	12.5%	/
Bo He [*Mentha haplocalyx* Briq.]	29.4%	33.3%	/	/
Bo Zi Ren [*Platyclatus orientalis* (L.) Franco]	/	/	50.0%	/
Chan Su [*Bufo bufo gargarizans* Cantor]	/	/	12.5%	/
Chai Hu [*Bupleurum chinense* DC.]	94.1%	77.8%	50.0%	83.3%
Cao Bai Zhu [*Atractylodes macrocephala* Koidz.]	5.6%	22.2%	/	/
Chen Pi [*Citrus reticulata* Blanco]	17.6%	33.3%	25.0%	33.3%
Chuan Xiong [*Ligusticum striatum* DC.]	17.6%	22.2%	25.0%	83.3%
Chi Wu Jia Pi [*Acanthopanax gracilistylus* W.W.Sm.]	/	/	12.5%	/
Dang Gui [*Angelica sinensis* (Oliv.) Diels]	64.7%	55.6%	12.5%	16.7%
Dan Pi [*Paeonia* × *suffruticosa* Andrews]	29.4%	11.1%	/	16.7%
Dan Shen [*Salvia miltiorrhiza* Bunge.]	11.8%	11.1%	25.0%	/
Da Zao [*Ziziphus jujuba* Mill.]	11.8%	/	50.0%	/
Fu Ling [*Poria Cocos* (Schw) Wolf.]	58.8%	88.9%	50.0%	33.3%
Fo Shou [*Citrus medica* L.]	/	22.2%	37.5%	/
Gan Cao [*Glycyrrhiza uralensis* Fisch.]	29.4%	22.2%	62.5%	33.3%
Gan Cao (Honey-toasted) [*Glycyrrhiza uralensis* Fisch.]	35.3%	33.3%	25.0%	16.7%
Gui Yuan [*Dimocarpus longan* Lour.]	/	/	37.5%	/
Gui Zhi [*Cinnamomum cassia* (L.) J.Presl]	/	/	12.5%	/
He Huan Hua [*Albizia julibrissin* Durazz.]	5.9%	/	/	16.7%
He Huan Pi [*Albizia julibrissin* Durazz.]	29.4%	22.2%	37.5%	50.0%
Huang Lian [*Coptis chinensis* Franch.]	/	/	12.5%	/
Huang Qin [*Scutellaria baicalensis* Georgi]	5.9%	11.1%	12.5%	/
Huang Qi (Honey-toasted) [*Astragalus membranaceus* (Fisch.) Bunge]	/	/	12.5%	/
Jiang [*Zingiber officinale* Roscoe]	23.5%	/	25.0%	/
Mai Dong [*Ophiopogon japonicus* (Thunb.) Ker Gawl.]	5.9%	/	12.5%	/
Mu Xiang [*Aucklandia lappa* DC.]	/	22.2%	12.5%	/
Niu Huang [Bos taurus domesticus Gmelin.]	/	/	12.5%	/
Qing Pi [*Citrus reticulata* Blanco]	/	11.1%	25.0%	33.3%
Ren Shen [*Panax ginseng* C.A.Mey.]	/	/	12.5%	/
Rou Gui [*Cinnamomum cassia* (L.) J.Presl]	/	/	12.5%	/
She Xiang [*Moschus berezovskii* Flerov]	/	/	12.5%	/
Di Huang [*Rehmannia glutinosa* (Gaertn.) DC.]	/	11.1%	12.5%	/
Shi Chang Pu [*Acorus tatarinowii* Schott]	41.2%	11.1%	/	/
Su He Xiang [*Liquidambar orientalis* Mill.]	/	/	12.5%	/
Tai Zi Shen [*Pseudostellaria heterophylla* (Miq.) Pax ex Pax et Hoffm.]	/	11.1%	12.5%	/
Xiang Fu [*Cyperus rotundus* L.]	23.5%	44.4%	/	83.3%
Xiao Mai [*Triticum aestivum* L.]	5.9%	/	25.0%	/
Ye Jiao Teng [*Reynoutria multiflora* (Thunb.) Moldenke]	5.9%	/	/	16.7%
Yu Jin [*Curcuma wenyujin* Y.H.Chen & C.Lin]	41.2%	11.1%	50.0%	100%
Yuan Zhi [*Polygala tenuifolia* Willd.]	23.5%	/	37.5%	16.7%
Zao Ren [*Ziziphus jujuba* var. *spinosa* (Bunge) Hu ex H.F.Chow]	35.3%	11.1%	25.0%	16.7%
Zhi Ke [*Citrus* × *aurantium* L.]	35.3%	66.7%	/	83.3%
Zhi Zi [*Gardenia jasminoides* J.Ellis]	41.2%	11.1%	/	16.7%

^a^Individual herbs used in at least 10% of the studies on a particular TCM pattern were listed.

**Table 3:** Effective rate and Hamilton Depression Rating Scale (HAMD) score of pattern-based Chinese herbal medicine treatment for depression.

Chinese herbal formula	Type of cases	Mean effective rate in % (range, 95% CI)	Mean HAMD change score (range, 95% CI)
*Liver qi depression *			
Xiaoyao decoction/pill (*N* = 4)	Depression (*N* = 4)	84.7 (70.0–93.3, 80.8–84.7)	19.1 (12.8–26.0, 18.4–19.9)
Chaihu Shugan decoction (*N* = 2)	Depression (*N* = 1); poststroke depression (*N* = 1)	90.0 (86.7–93.3, 88.8–91.2)	15.0 (14.6–15.4, 14.9–15.1)
Jieyu Heji (*N* = 1)	Depression (*N* = 1)	51.0 (NA)	NR
Yushu pill (*N* = 1)	Depression (*N* = 1)	74.3 (NA)	NR
All (*N* = 8)		85.1 (51.0–93.3, 77.3–80.9)	17.7 (12.8–26.0, 17.2–18.63)
*Liver depression and spleen deficiency *			
Xiaoyao decoction/pill (*N* = 1)	Depression comorbid diabetes (*N* = 1)	NR	7.3 (NA)
Yiqiyangyin Shuganjieyu decoction (*N* = 1)	Depression comorbid diabetes (*N* = 1)	83.3 (NA)	12.1 (NA)
Chaihu Shugan decoction (*N* = 1)	Depression (*N* = 1)	92.1 (NA)	NR
All (*N* = 3)		87.7 (83.3–92.1, 86.2–89.2)	9.7 (7.3–12.1, 8.9–10.5)
*Dual deficiency of the heart and spleen *			
Guipi decoction (*N* = 1)	Depression (*N* = 1)	NR	NR
Xiaochaihu Tang (*N* = 1)	Depression (*N* = 1)	NR	NR
Anshen Jieyu Fang decoction (*N* = 1)	Depression comorbid diabetes (*N* = 1)	NR	10.7 (NA)
Jieyu Yi Hao (*N* = 1)	Depression (*N* = 1)	90.0 (NA)	11.4 (NA)
Shexiang Baoxin pill (*N* = 1)	Depression (*N* = 1)	92.2 (NA)	NR
All (*N* = 5)		91.5 (90.0–92.2, 91.3–91.6)	10.8 (10.7–11.4, 10.6–11.0)
*Liver depression and qi stagnation *			
Chaihu Shugan decoction (*N* = 2)	Depression (*N* = 2)	89.8 (86.7–93.0, 88.8–90.9)	17.3 (13.9–20.7, 16.2–18.4)
Shujiele Wutang pill (*N* = 1)	Depression (*N* = 1)	91.4 (NA)	18 (NA)
All (*N* = 3)		90.4 (86.7–93.0, 89.7–91.0)	17.5 (13.9–20.7, 16.9–18.2)
All studies (*N* = 18)^ a^		85.0 (51.0–93.33, 84.1–85.9)	15.4 (7.3–26.0, 15.0–15.8)

^a^One study studied more than one TCM pattern; NA: not applicable; NR: not reported.

**Table 4:** Effective rate and Hamilton Depression Rating Scale (HAMD) score of combined pattern-based Chinese herbal medicine treatment and antidepressants for depression.

Chinese herbal formula	Type of cases	Mean effective rate in % (range, SD, 95% CI)	Mean HAMD change score (range, 95% CI)
*Liver qi depression *			
Xiaoyao decoction/pill (*N* = 9)	Depression (*N* = 6); poststroke depression (*N* = 3)	86.0 (70.0–94.3, 85.1–87.0)	19.1 (11.4–23.2, 18.4–19.9)
Banxia Houpu decoction (*N* = 1)	Depression (*N* = 1)	97.2 (NA)	16.0 (NA)
Kaiyu Quhuo decoction (*N* = 1)	Depression (*N* = 1)	100 (NA)	NR
All (*N* = 11)		88.3 (70.0–100.0, 87.4–89.2)	17.8 (11.4–23.2, 17.5–18.2)
*Liver depression and spleen deficiency *			
Xiaoyao decoction/pill (*N* = 8)	Depression (*N* = 8)	86.0 (66.7–95.1, 84.6–87.4)	21.5 (20.4–23.0, 21.3–21.7)
Self-invented formula (*N* = 1)	Depression (*N* = 1)	91.7 (NA)	NR
Kuaiwei Shugan pill (*N* = 1)	Depression (*N* = 1)	NR	12.2 (NA)
All (*N* = 10)		86.8 (66.7–95.1, 85.6–88.0)	20.1 (12.2–23.0, 19.6–20.5)
*Dual deficiency of the heart and spleen *			
Guipi decoction (*N* = 1)	Depression comorbid diabetes (*N* = 1)	92.1 (NA)	NR
Ningcao Wangyou decoction (*N* = 2)	Depression (*N* = 2)	53.8 (NA)^a^	16.8 (NA)^a^
Ganmai Dazao decoction plus Suanzaoren decoction (*N* = 1)	Poststroke depression (*N* = 1)	100.0 (NA)	6.0 (NA)
All (*N* = 4)		77.0 (53.8–100.0, 68.7–85.2)	11.4 (6.0–16.8, 9.5–13.3)
*Liver depression and qi stagnation *			
Chaihu Shugan decoction (*N* = 3)	Depression (*N* = 2); poststroke depression (*N* = 1)	80.0 (NA)^a^	15.0 (8.9–21.1, 12.8–17.1)
Xiaoyao decoction/pill (*N* = 2)	Depression (*N* = 2)	NR	NR
All (*N* = 5)		80.0 (NA)	15.0 (8.9–21.1, 12.8–17.1)
All studies (*N* = 24)^b^		86.3 (53.8–100.0, 85.5–87.2)	17.7 (6.0–22.6, 17.4–18.0)

^a^Only one study presented the data; ^b^five studies studied more than one TCM pattern; NA: not applicable; NR: not reported.
